# Preparedness and management of global public health threats at points of entry in Ireland and the EU in the context of a potential Brexit 

**DOI:** 10.1186/s12992-019-0496-4

**Published:** 2019-09-03

**Authors:** Máirín Boland, Mary O’Riordan

**Affiliations:** 1 Department of Public Health, Health Services Executive East, Dr. Steevens Hospital, Dublin 8, Ireland; 2 Health Protection Surveillance Centre, 25-27 Middle Gardiner St, Dublin 2, Ireland

**Keywords:** Cross border, Point of entry, Port health, International health regulations, Brexit, Health security, Preparedness, Response, Port, Airport, Ground crossing, Council decision

## Abstract

Health security in the European Union (EU) aims to protect citizens from serious threats to health such as biological agents and infectious disease outbreaks- whether natural, intentional or accidental. Threats may include established infections, emerging diseases or chemical and radiological agents. Co-ordinated international efforts attempt to minimize risks and mitigate the spread of infectious disease across borders.

We review the current situation (March 2019) with respect to detection and management of serious human health threats across Irish borders- and what may change for Ireland if/when the United Kingdom (UK) withdraws from the EU (Brexit).

Specifically, this paper reviews international legislation covering health threats, and its national transposition; and EU legislation and processes, especially the relevant European Decision No. 1082/2013/EU of the European Parliament and of the Council on serious cross border threats to health with repeal of Decision No 2119/98/EC. We enumerate European surveillance systems and agencies which relate to port health security; we consider consortia and academic arrangements within the EU framework and established collaboration with the World Health Organization. We describe current Health Services Executive port health structures in Ireland which address preparedness and management of human health threats at points of entry. We appraise risks which Brexit could bring, reviewing literature on shared concerns about these risks, and we evaluate post-Brexit challenges for the EU, and potential opportunities to remain within current structures in shared health threat preparedness and response.

It is imperative that the UK, Ireland and the EU work together to mitigate these risks using some agreed joint coordination mechanisms for a robust, harmonised approach to global public health threats at points of entry.

## Background

### Brexit

Following a referendum held on 23 June 2016 in the United Kingdom (UK) which supported it leaving the EU, Article 50 of the Treaty on European Union (EU) was invoked. This started a two-year process which was due to conclude with the UK’s exit on 29 March 2019, which at the time of writing has been extended until October 2019. This process is known as “Brexit”. Optimal preparedness for, and response to, international imported health threats requires surveillance, with robust early warning and response mechanisms which are shared across nations. In the context of Brexit, there are concerns about the maintenance of the current high degree of commitment to those systems and policies within the EU which have been firmly established and are multi-lateral in nature and which act to prevent, detect and manage serious infectious health threats spreading to Ireland and all EU states from elsewhere.

### International health regulations

The revised World Health Organization’s (WHO) International Health Regulations (2005) (IHR) [1] form an international legally binding instrument for 196 countries including Ireland and the UK and all other current EU Member States (MS). Following the global experience with SARS in 2003, IHR were seen to be in need of modification, and a revision of the 1969 IHR was adopted in 2005. They require countries to develop, strengthen and share public health information and to maintain capacities to prepare for, detect and respond to an international health threat at points of entry. IHR will continue to be binding for the UK under any Brexit.

### EU frameworks

In 2013 the European parliament and its 28 MS agreed on a legal framework on serious cross border threats to health. EU Decision 1082/2013/EU [2] addresses the all-hazards approach to emergency preparedness and response, to cover all public health threats, preparedness and response planning, ensuring interoperability between health, food, and veterinary sectors, as well as preparedness planning between nations. Systems enhanced under this decision include the EU Early Warning and Response System (EWRS); and European Centre for Disease Prevention and Control (ECDC) networks; and there are several EU agencies enhancing cross border protection at points of entry. The EU Decision aimed to further enhance IHR implementation in Europe [3].

This position paper from Ireland’s Medical Officer of Health Port Health group aims to review the current situation (March 2019) with respect to detection and management of serious human health threats across Irish borders and what may change for Ireland and the EU if/when Brexit occurs. We aim to describe the risks and challenges this would bring, and discuss potential models that have been used elsewhere to enhance interoperability across states neighboring the EU.

### Border structures

The Republic of Ireland, on the western edge of the EU, shares an island with Northern Ireland which is part of the UK, adjoining across an ‘invisible border’ of some 500 km. The existence of the Common Travel Area agreement since 1925 has permitted free movement of people on the island (i.e. no passport requirements). There has been neither border physical infrastructure nor border systems in place for some 20 years now, with an ‘invisible border’ since the 1998 Belfast/ ‘Good Friday’ agreement. The nature of any possible future land frontier between the EU and the UK in Northern Ireland has not been fully clarified, and it is a high priority for Ireland that any U.K. withdrawal from the EU would be negotiated to retain a porous non-structural border and to avoid a hard, structural border [4]. The draft withdrawal agreement for Brexit of July 2018 refers to the absence of a hard border in Northern Ireland on a permanent footing. The European Commission [5] acknowledges that different arrangements exist under the Common Travel Area between the UK and Ireland compared to other EU countries.

## Established cross border health security mechanisms

### International health regulations and their national transposition

IHR aim: ‘to prevent, protect against, control and provide a public health response to the international spread of disease in ways that are commensurate with and restricted to public health risks, and which avoid unnecessary interference with international traffic and trade’ [1].

The IHR have been transposed into national legislation in the jurisdictions of Ireland and the UK. Each country formally designates points of entry and ensures they have capacity at that point to detect and manage health threats.

The IHR cover all hazards, not just infectious diseases. The revised Regulations include a list of four diseases – smallpox, polio, Severe Acute Respiratory Syndrome (SARS) and new strains of human influenza – whose occurrence MS must immediately report to the WHO [1]. The Regulations provide a decision algorithm to determine whether other incidents, including those of a biological, chemical, radiological, or nuclear nature, may constitute a Public Health Emergency of International Concern (PHEIC). The WHO defines a PHEIC as, “an extraordinary event which is determined (i) to constitute a public health risk to other states through the international spread of disease and (ii) to potentially require a coordinated international response” [1]. The IHR also provide specific procedures and timelines for reporting and responding to these events.

The IHR were transposed into national legislation in Ireland via the Infectious Disease (Shipping) Regulations S.I. 4/2008 [6] and the Infectious Disease (Aircraft) Regulations S.I. 411/2009 [7]. While both sets of Regulations outline a schedule of serious infectious diseases to which they apply, including existing, re-emerging (e.g. plague) and novel diseases, this schedule also provides for the inclusion of unspecified, novel threats of international importance, should they arise.

In terms of points of entry, currently Ireland has three IHR designated airports (Dublin, Shannon and Cork) [7] and five IHR designated sea ports (Dublin, Cork, Limerick, Waterford, Rosslare) [6], all of which are required under IHR to maintain core capacities to deal with possible cross border health threats. There is no ground crossing designated under IHR as there is an ‘invisible border’ between Northern Ireland and Ireland.

The binding nature of IHR [1] and the essence of national legislation transposing these regulations will not be affected by any Brexit. However ground crossing designation, if any, will have repercussions for Ireland and the EU which are discussed later.

### Health security mechanisms established under the EU

There are a number of health security measures established under the EU including:

I. EU Decision 1082/ 2013/EU [2];
II. Systems, networks and agencies enhanced under this decision including the ECDC and the EU EWRS; the European Maritime Safety Agency (EMSA) and the European Aviation Safety Agency (EASA).
III. EU consortia and initiatives.

We describe these below and allude to possible consequences in case of Brexit.

I. Decision EU 1082/2013/EU

Decision EU 1082/ 2013/ EU on serious cross border threats provides mechanisms and tools to manage health crises in the EU, facilitating communication and co-ordination between EU MS in response to all types of hazards: biological, biotoxins, environmental, chemical [2]. It allows EU MS to coordinate plans, ensuring consistency and best practice sharing. Under this decision MS share procurement of medical countermeasures such as vaccines or medicines. Under EU 1082 there is established surveillance of over 40 infectious diseases across the EU, and the commencement of ad-hoc monitoring when chemical, environmental or biotoxin events occur.

The EU Health Security Committee (HSC), was set up in 2001 at the request of EU Health Ministers as an informal advisory group on health security at European level, and was formalized and strengthened by Decision 1082/2013/EU [2]. The HSC is mandated to reinforce the coordination and sharing of best practice and information on national preparedness activities, and to guide risk assessment, advice and crisis communication during an outbreak. MS also consult each other within the HSC with a view to coordinating national responses to serious cross border threats to health, including events declared a PHEIC. Following a Brexit, in the absence of arrangements, the UK will not be a full member of the EU HSC.

II. Systems, networks and agencies enhanced under EU 1082

The Network for the Surveillance and Control of Communicable Diseases was established by the European Parliament and Council Decision 2119/98/EC [8], superseded by Decision 1082/2013/EU [2]. The aim of the Network is to promote cooperation and coordination between the EU MS, with the assistance of the European Commission, with a view to improving the prevention and control of communicable diseases in the Community.

The European Centre for Disease Control (ECDC) coordinates 17 operational disease networks and supports several sub-networks of public health microbiology laboratories, to optimize pathogen detection, characterization and surveillance of specific diseases and antimicrobial resistance. These sub-networks integrate the epidemiological and microbiological surveillance of the EU notifiable communicable diseases listed in Decision 1082/2013/EU [2] on serious cross-border threats to health [9].

The EU has developed a strong operational early warning system, whereby under the EWRS MS have an obligation to report certain communicable disease information to the European Community. Ireland has formally signed up to EWRS via legislation [9]. The EWRS gathers such information from EU MS and issues alerts to the European Commission, EU MS and WHO. While operated by ECDC, it is controlled by the European Commission. The EWRS platform is being re-engineered to link with other EU alert and information systems [10]. A non-exhaustive list of alert and information systems at Union level to be progressively linked with EWRS is set out in the Annex to Commission Implementing Decision (EU) 2017/253 [10] laying down procedures for the notification of alerts as part of the EWRS and for the information exchange, consultation and coordination of responses to such threats pursuant to Decision 1082/2013 EU.

Risk assessment of a health threat that may affect EU MS is carried out by European agencies- such as ECDC, or the European Food Safety Authority (EFSA), with the input of expert Scientific Committees to advise on possible measures to take. Possible ECDC actions include preparing or updating a rapid risk assessment, continued monitoring of the event, launching an urgent inquiry, preparing an epidemiological update, posting a news item on the ECDC website, offering technical assistance to the affected Member State, and sharing information through the Epidemic Intelligence Information System (EPIS) platform [11]. EPIS is a web-based communication platform that allows nominated public health experts to exchange technical information to assess whether current and emerging public health threats have a potential impact in the EU.

There has been concern that the UK will not be able to secure a future partnership agreement with EU bodies such as the ECDC in a timely way [12]. Potential solutions are reviewed within the discussion.

The European Maritime Safety Agency (EMSA) includes the platform SafeSeaNet (EU ship monitoring), commercial information and geo-algorithms to locate ships (emsa.europa.eu), giving ports of call, dates of arrival and is of benefit in giving information regarding conveyances travelling from an affected country. With Brexit, the UK will no longer have access to SafeSeaNet, although the UK could obtain data independently through satellite providers and could contribute data voluntarily to the programme [13].

The Maritime National Single Window prototype system of the European Commission was launched by EMSA*.* It covers the information flows between the ship data providers (e.g. ship agent, master, shipping company), the relevant public authorities covering the port of call, and other EU MS via SafeSeaNet. The Maritime Declarations of Health noting and describing illness on board can be quickly and confidentially shared between countries with this system. Following Brexit, the UK will no longer participate in the Maritime National Single Window (NSW) [14]. Depending on their national legislation the UK could still independently operate a NSW, however the information-sharing aspect between and with EU countries would be affected.

The European Aviation Safety Agency (EASA) (Public Health Emergencies in Aviation) under EU 139/2014 [15] requires an emergency plan to be in place for an aerodrome, and produces relevant safety information bulletins (e.g. Ebola risk communication for airport operators), and works with the International Civil Aviation Authority (ICAO) which is a United Nations agency. The UK could still implement those plans and procedures after Brexit. However the networking with EU MS would be affected and the information and guidance could potentially not be distributed or availed of.

III. European consortia and initiatives

A number of EU initiatives have developed knowledge and guidance, literature and evidence-based reviews, and tested preparedness and response at points of entry (ShipSAN [16], AirSAN [17]).

The Joint Action Preparedness and action at points of entry (ports, airports, and ground crossings) known as Healthy Gateways [18] was prepared under the framework of the 2017 Annual Work Programme and has received funding from the EU, in the framework of the Third Health Programme (2014–2020). The Joint Action will produce guidelines, catalogues of best practices and validated action plans to be implemented by Member State health authorities at operational level in the field of transport, covering all types of health threats, risk communication, advice for public health event management and contingency planning. The action will also support rapid exchange of information in the event of cross-border health risks, using electronic means via established communication networks for points of entry.

In the event of future public health emergencies of international concern (PHEIC), the Joint Action will move from its inter-epidemic mode to an emergency mode, in order to support the coherent response of EU MS according to Decision No. 1082/2013/EU and implementation of temporary recommendations issued by the WHO according to IHR.

It is reassuring for the EU MS that collaboration between WHO/Europe, the European Commission (EC), the European Centre for Disease Prevention and Control (ECDC) and other relevant EU agencies on health security is already well established [11]. Countries within WHO Europe collaborate on event management within the WHO Europe region, and with EWRS [11]. The EU Health Security Committee may involve WHO or expert groups in its deliberations. WHO are partners in many collaborations and training initiatives eg PagNET [19], ShipSAN [16], AirSAN [17] and Healthy Gateways [18]. Following any Brexit WHO-related activities will not be affected, so the potential adverse consequence for Ireland/ EU/ UK of reduced collaboration and communication will be lessened due to the presence of this parallel pathway.

## Irish structures for cross border port health security

In 2009 an IHR External Assessment Group Report made recommendations on port health preparedness in Ireland and the *HSE Medical Officer of Health (MOH) Port Health Committee* was established as part of the response. This committee consists of Specialists in Public Health Medicine, who act as the Medical Officer of Health designated under national infectious disease legislation and national transposition of IHR. It acts as a forum for sharing experience and knowledge regarding preparedness for, and response to, communicable disease incidents at points of entry, and organizes training and desktop exercises to test guidance.

The *Health Service Executive (HSE) National Port Health network* was established in 2010 following the H1N1 2009 pandemic, as part of the HSE Emerging Viral Threats Committee. The Network brings together disciplines within the health services, including Environmental Health Services, Public Health Medicine, National Ambulance Services, Health Surveillance and IHR National Focal Point and Emergency Planning as network members. It progresses multidisciplinary working in relation to Port Health in collaboration with relevant stakeholders such as the ports, airports, government Departments of Health and of Transport, Tourism and Sport. It develops guidance and tests it through tabletop or live exercises. The interoperability of these port health functions in Ireland has been described [20].

The Port Health Network has developed and tested national guidance for preparedness and response. Communication protocols have been established and disseminated. These groups worked closely during the PHEIC of Ebola virus disease to improve knowledge and awareness for points of entry authorities (ports and airports), to assist in early detection of potentially infected persons, to disseminate health information for travelers, to develop protocols for assessment and case management, to ensure infection prevention and control at points of entry and to assist in implementing WHO recommendations related to the management of Ebola Virus Disease [20]. The Network is a collaborating partner in the EU Healthy Gateways programme [18] which maintains links with non-EU MS through WHO Europe. This provides an opportunity for some alliance between Ireland and the UK separate to EU structures.

These groups work through and with designated points of entry in Ireland (airports and seaports). The current absence of any border or ground crossing between Ireland and Northern Ireland means ground crossing collaboration has not existed. This would change considerably in case of a Brexit with a border and any designated ground crossing.

## Discussion

The EU, including the UK, is considered to have set a high global standard for the protection of human, animal and plant health [21]. Clearly, managing serious in-country health threats with cross border potential is beneficial to all neighboring countries, and the formal support and structures at EU MS level that have developed through the recent PHEICs of H1N1 pandemic, Ebola and Zika continue to progress through initiatives and training efforts under the EC/EU and also WHO.

In terms of preparedness and response to a health threat, following Brexit, IHR would still apply. IHR preparedness and capacities for health emergencies would be overseen by WHO, as the UK continues as a member of WHO Europe; and the UK would continue to communicate with, contribute to and avail of expertise from networks via WHO. The UK has been to the forefront in terms of communication and sharing of knowledge under IHR [22]. Between 2012 and 2016, the UK notified 52 events to WHO under IHR, mostly concerning potential PHEICs in a UK territory; most were related to infectious diseases. A total of 218 contact tracing incidents were logged by the UK’s National Focal Point with WHO under IHR between 2012 and 2016. The countries the UK NFP communicated most frequently with were USA, Australia, France, Canada, Singapore and Spain [22].

Post-Brexit the IHR focal point could become the liaison point using the WHO Event Information System; this occurs in non-EU contact situations for Ireland currently. A downside is the lack of an IHR secure electronic portal; although there is work underway to develop a secure electronic IHR portal for all hazards.

The risk for Ireland and the EU is that, without provision, the formal collaboration through EU mechanisms, processes and agencies described above may be reduced post-Brexit. While existing informal communications and established shared approaches such as via IHR may mitigate against the loss of these formal mechanisms, their robustness and resilience are unknown; Brexit will test the rigor of the remaining informal and parallel channels by creating a setting which has not been tested before- we are entering uncharted waters, and considerable risks have been identified.

### Risks

In the absence of alternative arrangements, risks could emerge in the potentially reduced and delayed interconnectedness between the UK and EU MS.

It will cost the UK to maintain its standards; preparedness is expensive and may have low discernible outcome when it is successful. The many arrangements that will need to be rewritten upon Brexit make it concerning that health security will not be prioritized [21].

In the absence of the EWRS mechanism of data sharing, epidemic intelligence in the UK regarding health threats may not be shared in the optimal way. There is a potential, for example, that those encountering a health threat arising in Northern Ireland would be limited to using the IHR platform, channeled through London, which is more formal and slower. This could reduce the speed of communication and intelligence sharing, delaying risk assessment, planning and response such as urgent containment measures with the nearby communities and country (Ireland) and the EU. Lack of the established secure electronic EWRS platform to permit the exchange of personally identifiable information may complicate hazard event management, challenge data protection, and could slow response times for contact tracing, or measures such as recall of food products. Coordination of public health advice across borders to the public may be affected due to lack of information sharing and shared consensus on the best approach.

Others [12, 23–25] agree that without EU 1082, and in the absence of bilateral agreements among the two neighboring countries, there could be loss of compatibility of preparedness plans, reduced preparedness practice together; or less consistent shared guidance. Brexit would ‘weaken the capacity of all parties to respond effectively to cross-border health emergencies’ [12]. The Brexit Health Alliance [23] consider that the efforts to prevent the spread of infectious diseases could be under threat if the country leaves the EWRS. It calls for full membership of the HSC, though requiring some ‘flexibility and revisions to the current EU legislative framework’. Health protection experts within the UK felt it was very important to retain a working relationship with ECDC post-Brexit [24], with EWRS being their most highly ranked ECDC function. Many felt that their ability to manage future outbreaks post-Brexit would be weakened. Faculty of Public Health members considered it is vital that Public Health does not ‘slip off the radar’ during Brexit negotiations [24, 25].

Optimal input of other EU systems such as other ECDC networks, EMSA, EFSA, EASA, European Medicines Agency, would be lost to the UK after Brexit unless special arrangements are in place. The UK would be external to non-health European supports such as Europol, relevant in the event of malicious organized health threats, such as biotoxin exposures.

Recently the EC has proposed the creation of a Standard Operating Procedure for the ad hoc urgent exchange and sharing of medical countermeasures based on the EWRS [26]. Brexit could bring supply chain risks regarding procurement of pandemic countermeasures for the UK which may impact on pandemic planning, constituting a threat if the UK cannot contain or mitigate a pandemic [12].

The extent of the risk is dependent on the nature of any Brexit. Fahy et al. reviewed the risk to different health-related issues given three Brexit scenarios [27]. With a soft Brexit, much of the EU law would continue to apply in the UK, though without UK input and without European Court of Justice interpretation and enforcement. Formally EU law would no longer be a source of UK law but de facto much of it would continue to be so in return for EU market access. However, the practicalities of how European law would be transposed into UK domestic law are unclear. A hard Brexit would see a cease to funding streams from the EU and research collaborations under the EU framework [27]. This ‘loss of access to UK research and expertise could further undermine pandemic preparedness planning and response in Europe’ [12].

In a soft Brexit [27], it is likely that current arrangements may continue. Collaboration may stop with no legal framework to govern it in a failed Brexit; in a hard Brexit the external position is likely to undermine collaboration.

In the potential eventuality of a hard border, the Ireland/ Northern Ireland border would be the only land frontier between the EU and the UK. Ireland would in this scenario be similar to other countries at the edge of Europe such as Sweden, which has a land border with Norway (European Economic Area member) and to those EU MS surrounding Switzerland (not a European Economic Area member). A hard border between Northern Ireland and the Republic of Ireland would create ground crossing/s. Designated ground crossings under IHR are designated by countries themselves and this might occur in the scenario of a Northern Ireland hard border. The WHO is currently reviewing preparedness, risk assessment and management of health threats at ground crossings in the EU (personal communication N. Wang, WHO) and a focus on preparedness is expected. All designated ground crossings need to be prepared to deal with cross border IHR threats, and core capacities would need to be identified and provided for by both sides, with significant governance, organizational, legislative and resource implications for Ireland. Cross border health services access in the case of health threats is paramount [23] and would need to be provided for.

### Options and opportunities for preparedness and communication

It would be best for Ireland, the EU and the UK if, post Brexit, the UK was part of a strong system for timely and urgent sharing of information regarding emerging health threats and controls, response, and situation reports. There has been a wide call for a health security partnership with strong co-ordination between EU and UK in dealing with serious cross border health threats such as pandemics, and infectious disease threats, ideally by continued access for UK to ECDC systems [24].

Regarding cross border issues a Memorandum of Understanding in relation to health protection and health security is currently being developed between the UK and Ireland.

The British government has promised, under a Faculty of Public Health UK paper, ‘*an unequivocal guarantee that UK public health protections and standards will be the same or higher when (the UK) have left the EU; health partnerships with the EU will remain just as strong in the future; and that the (UK’s) public’s health will be at the epicenter of the UK’s Brexit negotiations and future policy making’* [28].

On foot of a UK withdrawal from the EU, existing mechanisms may be replaced or may be covered by other aspects of legislation, WHO agreements, repeal or memoranda of understanding [27]. For the UK to entirely leave EU structures and systems and continue to work via the WHO or UN Codex Alimentarius for epidemic surveillance will entail loss of influence [27]. A UK governmental paper sets out their ongoing work with key EU agencies on health security systems and infrastructure to enable information sharing and access to key datasets [21], acknowledging that infectious diseases can be geographical threats that require global responses. The UK proposes [21]: ‘*continuing close collaboration with the Health Security Committee and bodies such as ECDC, including access to all associated alert systems, databases and networks, to allow the UK and the EU MS to coordinate national responses; and collaboration with the European laboratory surveillance networks to monitor the spread of diseases across Europe’.*

A Faculty of Public Health (UK) paper explores the different options for cross-border health protection in the future, suggesting alternative models, which do exist ‘across the wider European neighborhood area’ [25]. The preferred option is retaining the current arrangement with ECDC (at a financial cost), followed by creation of a bespoke relationship with ECDC and other international health security organizations. Their least preferred option is creation of European Neighbourhood Policy agreements.

Provisions could be made for continuance through either UK application to be part of the EWRS, or the UK having a special arrangement to avail of the EWRS, or as part of a developed interoperable epidemic intelligence system. Norway, although not an EU MS but a member of the European Economic Area (EEA), [29] has made provision to be part of the EWRS. Norway participates in discussions of the ECDC at high level, and has access to EWRS, but has no decision-making authority.

The EEA Agreement of 1994 expands the EU’s single market to include Norway, Iceland and Liechtenstein. In practice, the Agreement allows people, goods, capital and services to circulate freely in the whole EEA Area. Whether the UK will be part of the EEA on Brexit is unclear currently. Switzerland, in neither the EU nor EEA, avails of a special arrangement via Lichtenstein for EWRS.

The Epi-South project of 2010, involving Mediterranean EU MS e.g. Italy, Malta plus some African Mediterranean States e.g. Morocco, Algeria, is one model for addressing epidemic intelligence and interoperability with EWRS [30].

The option of becoming an observer for the Health Security Council (such as currently Iceland, Liechtenstein, Norway, Turkey and Serbia) would mean that the UK would be party to the information exchange, but would not be a key participant.

One review suggests that there is little reason in theory why cooperation in information sharing could not continue, but this depends on financing and investment in collaboration and the UK being given ‘adequacy status’ under GDPR for sharing of personal information [27]. Many countries aspiring to be in the EU aspire to EU standards in health security management, for example Turkey as an accession state [31].

We highlight one particular constructive option that has been little discussed to date. Reviewing the preamble (26) to Decision 1082 [2], the potential does exist for continued EWRS participation for the UK if it is a ‘third country’ (non-EU, non-European Free Trade Association) in case of Brexit. *‘Within the limits of the Union’s competences, such agreements could include, where appropriate, the participation of such third countries or international organisations in the relevant epidemiological surveillance monitoring network and the EWRS, exchange of good practice in the areas of preparedness and response planning, public health risk assessment and collaboration on response coordination.*’ [2].

The aim of this preamble, presumably, is to be consistent with IHR obligations generally and for the benefit of EEA countries, in the interest of public health. The Decision states: ‘*It could be in the interests of the Union to conclude international cooperation agreements with third countries or international organisations, including the WHO, to foster the exchange of relevant information from monitoring and alerting systems on serious cross border threats to health.’*

We interpret this to mean that there is an opportunity for negotiation at EU level to provide the UK with third party access to the various systems and to share information with them.

While it is imperative that the communication channels and early warning systems are optimized, the operational components for Ireland’s points of entry, such as the HSE Port Health Network, maintain preparedness for a port heath threat irrespective of point of origin internationally, whether EU or non-EU. In case of Brexit, flights coming in from the UK to Ireland will be like flights from other non- EU jurisdictions. Public Health emergency protocols are in place and exercised at Ireland’s airports. Similarly ships coming in from the UK should not pose a significant difference in terms of health threat experience for Ireland, although non-EU membership brings additional checks and delays. Ireland has received freight ships directly from a country affected by a Public Health Emergency of International Concern (Guinea, Ebola Virus Disease) in the past and has managed human health threats via established and enhanced mechanisms [20].

## Conclusion

The MOH Port Health group considers a continued collaborative partnership for the UK and the EU within existing structures would be optimal for cross border health protection in Ireland, ideally with ECDC and EWRS membership continuance. The IHR platform provides one complementary modus operandi.

We acknowledge that informal networks and collaborations, well established between the UK and Ireland would remain a very important means of communication. Shared experiences since SARS in 2003 and through pandemic influenza in 2009 and Ebola in 2014/15 have built a strong network of contacts which must continue.

Our preparedness plans as they exist are not specific to threats entering from EU or non-EU countries and preparedness plans for all-hazards at current points of entry are not dependent on Brexit outcomes, except in the case of any further designated point of entry.

The MOH Port Health group considers and has advised that the memorandum of understanding between the UK and Ireland must prioritize health protection and cross-border health security. Communication and co-ordination is key.

We anticipate that Ireland, the EU and the UK will, in whatever form Brexit may take, be able under some creative formal EU-UK joint coordination mechanisms to continue to work harmoniously in a responsible shared approach to global health threats. It is imperative that processes to address cross border health protection concerns be addressed urgently and multilaterally as a possible Brexit draws closer.

## Data Availability

Not applicable.
